# Physical activity does not alter prolactin levels in post-menopausal women: results from a dose-response randomized controlled trial

**DOI:** 10.1186/s11556-017-0179-1

**Published:** 2017-07-13

**Authors:** Darren R. Brenner, Yibing Ruan, Andria R. Morielli, Kerry S. Courneya, Christine M. Friedenreich

**Affiliations:** 1 Department of Cancer Epidemiology and Prevention Research, CancerControl Alberta, Alberta Health Services, Holy Cross Centre, Room 513C, Box ACB, 2210 - 2nd St. SW, Calgary, AB T2S 3C3 Canada; 2 Department of Oncology, Cumming School of Medicine University of Calgary, Calgary, AB Canada; 3 Department of Community Health Sciences, Cumming School of Medicine University of Calgary, Calgary, AB Canada; 4 Faculty of Physical Education and Recreation University of Alberta, Edmonton, AB Canada

**Keywords:** Physical Activity, Randomized Controlled Trial, Breast Cancer, Prolactin

## Abstract

**Background:**

Increased circulating levels of prolactin have been associated with increased risk of both in situ and invasive breast cancer. We investigated whether or not physical activity had a dose–response effect in lowering plasma levels of prolactin in postmenopausal women.

**Methods:**

Four hundred previously inactive but healthy postmenopausal women aged 50–74 years of age were randomized to 150 or 300 min per week of aerobic physical activity in a year-long intervention. Prolactin was measured from fasting samples with a custom-plex multiplex assay.

**Results:**

A high compared to moderate volume of physical activity did not reduce plasma prolactin levels in intention-to-treat (Treatment Effect Ratio (TER) 1.00, 95% Confidence Interval (CI) 0.95 – 1.06) or per-protocol analyses (TER 1.02, 95% CI 0.93 – 1.13).

**Conclusions:**

It is unlikely that changes in prolactin levels mediate the reduced risk of breast cancer development in post-menopausal women associated with increased levels of physical activity.

**Trial registration:**

clinicaltrials.gov identifier: NCT01435005.

## Introduction

The primary hypothesized mechanisms underlying the associations between physical activity and reduced breast cancer include reductions in adiposity, sex hormone levels, insulin resistance and chronic inflammation [1]. These pathways only explain some portion of the association and additional mechanisms require identification and further investigation.

Prolactin is a luteotropic peptide hormone involved in regular lactation which is produced by the anterior pituitary gland. Increased circulating levels of prolactin have been associated with increased risk of both in situ and invasive breast cancer [2]. Two previous exercise intervention trials in post-menopausal women did not observe changes in prolactin levels in response to moderate physical activity [3, 4] compared to controls. As part of the Breast cancer and Exercise Trial in Alberta (BETA) we investigated: 1) the effects of increased levels of moderate to vigorous physical activity (MVPA) on levels of prolactin and 2) whether a higher level of activity led to larger changes in prolactin levels.

## Materials and methods

The design of the BETA study has been previously described in detail [5]. Briefly, 400 previously inactive but otherwise healthy postmenopausal women of age were randomized to 150 (MODERATE) or 300 (HIGH) minutes per week of aerobic physical activity for a year-long intervention. Women were eligible for randomization if they were between 50–74 years of age, with no previous diagnosis of invasive cancer, no major comorbidities, obtained physical approval for participation, had a body mass index of 22–40, were moderately sedentary, not a current smoker or excessive drinker, not currently on a weight loss program, English speaking and not planning to be out of the study site areas for more than 4 consecutive weeks during the subsequent 18 months. Both intervention arms were prescribed the same frequency (five days/week) and intensity (moderate-to-vigorous) of aerobic exercise. The training targets were 60 minutes/session (300 total minutes/week) for the HIGH group, and 30 minutes/session (150 total minutes/week) for the MODERATE group. Wrist-worn heart rate were given to each participant and used to ensure a 65–75% maximum heart rate reserve (HRR) was being achieved during each exercise session. A ramp-up period was included where the intensity, frequency and duration of exercise were gradually increased during the first three months of the intervention until the target exercise prescriptions were attained. Fasting blood samples were collected from all participants at baseline, 6 and 12 months following a 24 h abstinence from alcohol intake and exercise and at least 10 h after their last meal. Prolactin levels in plasma were measured with a custom-plex multiplex assay (Eve Technologies, Calgary, AB, Canada), using the Bio-Plex™ 200 system (Bio-Rad Laboratories, Inc., Hercules, CA, USA). The assay sensitivity for prolactin was 30.2 pg/ml and the inter-assay coefficient of variation was 5.5%. Samples for each participant at different time points were all analyzed in the same batch and each batch included an equal number of MODERATE and HIGH blood samples.

Predicted VO_2_ max was estimated from a modified Balke treadmill test [6] using the multistage model and the American College of Sports Medicine metabolic equations for estimating maximum oxygen consumption [7]. Body composition was estimated from full body dual energy X-ray absorptiometry (DXA) scans to assess overall percent body fat and total fat mass. Computed tomography (CT) scans were taken at the level of the umbilicus to measure subcutaneous and intra-abdominal adiposity.

We considered the comparison of means of 12-month outcomes (log transformed, with no adjustment for baseline values) for the prolactin outcomes. Standard deviation was estimated from previous reports [8]. A sample size of 200 participants per group provided 90% power to detect anticipated changes of 4% between the treatment groups at α = 0.05. Prolactin changes in the two arms were compared in both intention-to-treat and per-protocol analysis using linear mixed models as previously described, adjusting for baseline prolactin levels [9]. A per-protocol analysis was conducted on participants achieving ≥60% of prescribed exercise duration in their target heart rate zone. To investigate whether the effects of exercise were restricted to particular subgroups, stratified analyses were conducted on *a priori* variables including baseline body mass index (BMI (weight (kg)/height (m^2^)), estimated physical fitness (VO_2max)_, and total percent body fat.

The study protocol was approved by the Alberta Cancer Research Ethics Committee and the Conjoint Health Research Ethics Board of the University of Calgary and the Health Research Ethics Board of the University of Alberta. All participants provided written informed consent.

## Results

The distribution of the study participants’ baseline characteristics was similar in the two trial arms with no meaningful differences between arms [9]. Baseline prolactin levels were 10565 (SD = 6963) pg/ml in MODERATE group and 10478 (SD = 5150) pg/ml in HIGH group (*p* = 0.89). Overall, we did not observe statistically significant effects for increasing quartiles of physical activity and change in level of prolactin in the *n* = 384 women included in the analyses (non-randomized analysis – results not shown).

No statistically significant differences were observed in the treatment effect ratios of the two exercise groups in either intention-to-treat (TER 1.00, 95% CI 0.95 – 1.06) or per-protocol analyses (TER 1.02, 95% CI 0.93 – 1.13; Table 1). A per-protocol analysis examining the role of exercise intensity (< or ≥60% of prescribed exercise) also showed no change in prolactin levels between the two groups (Table 1). In stratified analyses by estimated physical fitness (VO_2max_) and total body fat, no treatment effect ratios in any of the subgroups were significantly different from 1.0 (Table 2).

**Table 1:** Intention-to-treat and per-protocol analyses of prolactin concentrations between high and moderate volume exercise groups in BETA, (*n* = 386)

Group	n	Baseline		6 Months		12 Months		Treatment effect		Between-group P^c^
		Geometric Mean^a^	95% CI	Geometric Mean	95% CI	Geometric Mean	95% CI	Ratio of High/Moderate^b^	95% CI	
Intention-to-treat										0.90
High	195	9368	8720 – 10064	9353	8741–10008	9035	8467 – 9641	1.00	0.95 – 1.06	
Moderate	191	9331	8713 – 9992	9230	8644–9855	9017	8457 – 9613			
Per-protocol^d^										0.62
High	80	8761	8002 – 9591	9158	8225–10198	8700	7904 – 9576	1.02	0.93 – 1.13	
Moderate	58	9653	8284 – 11248	9251	8110–10551	9366	8275 – 10600			

^a^Geometric mean of prolactin was in the unit of pg/mL

^b^The geometric mean ratios were estimated from least square means for the difference in treatment effect between high and moderate volume exercisers averaged across the entire study period adjusted for the baseline values and then back log-transformed

^c^ *P* value corresponds to the null hypothesis that the ratio of treatment effect between high- and moderate-volume groups equals 1 against the 2-sided alternative hypothesis

^d^Women assigned to the moderate-volume group were adherent if they completed 90% to 100% of the exercise prescription (mean, 135–150 min/week), weeks 13 to 52 at full prescription; women assigned to the high-volume group were adherent if they completed at least 90% of the exercise prescription (mean, ≥270 min/week), weeks 13 to 52 at full prescription

**Table 2:** Stratified analyses of changes in prolactin between high volume and moderate volume exercise groups in BETA, 2010–2013 (*n* = 386)

Stratification variables	Prescribed exercise duration	n	6 months percent change from baseline	12 months percent change from baseline	Treatment effect^a^		Between-group P^b^
					Ratio of HIGH:MOD	95% CI	
Time at high intensity^c^							
<60% prescribed	MODERATE	17	−2.0	−6.4	1.06	0.93 – 1.21	0.40
	HIGH	44	8.0	−0.3			
≥60% prescribed	MODERATE	41	−5.1	−1.5	1.01	0.88 – 1.17	0.85
	HIGH	36	0.5	−1.2			
BMI^d^							
< 30	MODERATE	116	15.1	16.0	0.98	0.91, 1.06	0.59
	HIGH	121	−4.7	−4.7			
≥ 30	MODERATE	75	11.3	3.6	1.05	0.95, 1.15	0.36
	HIGH	74	7.7	−1.6			
VO_2max_ level^e^							
< 27.2 ml/kg min	MODERATE	93	12.7	2.5	1.04	0.96, 1.13	0.38
	HIGH	98	3.7	−2.2			
≥ 27.2 ml/kg min	MODERATE	98	14.4	19.8	0.98	0.90, 1.06	0.57
	HIGH	97	−3.9	−5.0			
Total body fat^f^							
< 29.7 kg	MODERATE	94	9.1	8.9	0.98	0.90, 1.07	0.61
	HIGH	99	−4.7	−3.8			
≥ 29.7 kg	MODERATE	97	18.1	13.0	1.03	0.95, 1.12	0.48
	HIGH	96	4.7	−3.3			

^a^HIGH:MODERATE ratio of geometric means for prolactin levels over 12 months adjusted for prolactin level at baseline. A ratio <1.0 indicates lower prolactin concentrations in the HIGH exercise group at 6 and 12 months; a ratio >1.0 indicates lower prolactin concentrations in the MODERATE exercise group; a ratio equal to 1.0 indicates no difference in prolactin concentrations between the HIGH and MODERATE exercise groups

^b^P for testing the HIGH-MODERATE group difference over 12 months from the linear mixed model, adjusted for prolactin level at baseline

^c^Included n = 138 women for whom, across weeks 13–52 (at full prescription), average adherence in the exercise logs was 90–100% in the MODERATE group (135–150 min/week; n = 58) or ≥90% in the HIGH group (≥270 min/week; n = 80). Time at high intensity was defined as time exercising at an intensity of 60–80% heart rate reserve averaged for each participant over 52 weeks. Cut points for the stratified analysis were 60% of the prescribed durations, i.e., 90 min/week in the MODERATE group and 180 min/week in the HIGH group

^d^Body mass index at baseline

^e^Cut point of the baseline VO_2max_ level for the stratified analysis were the median value for all study participants (MODERATE and HIGH group)

^f^Cut point of the baseline total body fat level for the stratified analysis were the median value for all study participants (MODERATE and HIGH group)

## Discussion

Overall, a higher volume of exercise compared to a standard volume did not reduce prolactin levels in BETA. Moreover, the effects did not vary according to exercise adherence or baseline fitness levels, BMI, and total body fat.

This exercise intervention trial is the first study to examine the effects of a high versus moderate volume of MVPA aerobic exercise on prolactin levels in postmenopausal women. Previous studies comparing 12 months of moderate-intensity aerobic exercise to no exercise have reported null effects on prolactin levels [3, 4]. Similarly, the results from these previous trials remained unchanged when exercise adherence, measured as minutes of exercise per day, was considered [3, 4]. In the study by Reding et al. [4], baseline BMI did not alter the treatment effect. In the study by Tworoger et al. [3], change in percent body fat did not mediate the intervention effect however, women in the exercise group who increased their VO_2 max_ by >5% had a statistically significant reduction in prolactin levels.

Our analyses were motivated by the strong animal and in vitro data supporting an important role of prolactin in breast carcinogenesis [10] and epidemiologic data suggesting an association of increased levels with breast cancer risk [11]. While our study did not observe significant impacts of exercise on levels of prolactin, it is worth noting that there are complex relationships between exercise and prolactin levels which may explain our null findings. For example, threshold effects of exercise intensity have been observed in the literature [12], which suggests that the intensity of exercise in the BETA trial may not have been high enough despite a target >65% HRR. Furthermore, effects may differ by age as there are several studies among young athletic populations where increased levels of prolactin are reported post-acute bouts of exercise [12–14], while effects among older sedentary populations are less well characterized.

In conclusion, the results from the BETA study suggest that 300 min per week of moderate-intensity aerobic exercise does not reduce prolactin levels more than 150 min per week in a year-long intervention in postmenopausal women. It is unlikely that changes in prolactin levels mediate the reduced risk of breast cancer development in post-menopausal women associated with increased physical activity at any level.
